# Feed-forward Control Nursing Model in Expectant Treatment of Placenta Previa

**Published:** 2017-02

**Authors:** Yanfei ZHU, Shuxuan ZHANG, Wenxian SHAN, Ming HU

**Affiliations:** 1. Dept. of Obstetrics, The First People’s Hospital of Xuzhou, Xuzhou, 221002, China; 2. Dept. of Nursing, The First People’s Hospital of Xuzhou, Xuzhou, 221002, China

**Keywords:** Feed-forward control nursing, Placenta previa, Expectant treatment, Specialist nursing

## Abstract

**Background:**

We studied the possible advantages of feed-forward control nursing model in the treatment of placenta previa.

**Methods:**

We enrolled 60 pregnant women who were receiving treatment for expectant placenta previa between January 2010 and January 2016 and randomly divided them into the control group and the observation group with 30 cases in each group. In the control group, we offered specialist nursing which included examination, body positioning, vaginal bleeding record, psychological consultation and medication observation. Feed-forward control nursing was applied in the observation group which included establishing feed-forward control nursing improvement team, conducting quality control of nursing defects and putting forward ideas for improvements and verifying improvement outcomes.

**Results:**

The observation group got significantly higher success rate and lower complication rate compared with control group. Gestational age and fetal weights improved apparently in the observation group. When we compared the amount of postpartum bleeding and pregnancy bleeding in two groups we did not find any statistically significant difference (*P*>0.05). Patients’ satisfaction rate toward our nursing services was much higher in the observation group and the rate of nursing errors was significantly lower in this group. All differences were statistically significant (*P*<0.05).

**Conclusion:**

Application of feed-forward control nursing model in the expectant treatment of placenta previa can improve treatment success rate, decrease complications and upgrade nursing quality.

## Introduction

Placenta previa is the most common cause of bleeding during the third trimester, which can be linked to endometrial lesions of uterine body, over-sized placenta, placental abnormality and delayed development of Zygote trophoblast. Placenta previa’s incidence rate is approximately 0.3% to 1.8% and can severely threaten the health of the mother and her baby (1).

Expectant treatment is a kind of selection method, which is safer than other methods and offers the most favorable pregnancy outcomes for mother and baby after comprehensive evaluation towards the condition of pregnancy age less than 36 weeks, fetal weight less than 2.3 kg, few vaginal bleeding, pregnant woman in good body condition and fetal survival (2). During the treatment, use of scientific and cautious nursing techniques can make a huge difference (3).

Feed-forward control nursing model fills the existing gaps and defects in the routine specialist nursing, and puts forward practical measures to improve the quality of the care given to patients (4). This model reflects various ideas, combines them with specific pregnancy condition, and evaluates the possible adverse conditions, which may appear during the expectant treatment (5).

In this research, we studied the advantages of feed-forward control nursing model in the expectant treatment of placenta previa.

## Material and Methods

### Patients’ data

From January 2010 to January 2016, we enrolled 60 pregnant women who were receiving treatment for expectant placenta previa and randomly divided them into the control group and the observation group with 30 cases in each group. In the control group, we offered specialist nursing which included examination, body position, vaginal bleeding record, psychological consultation and medication observation. Feed-forward control nursing was used in the observation group including establishing feed-forward control nursing improvement team, conducting quality control of nursing defects and putting forward improvement idea and checking improvement outcome.

This study was approved by the Ethics Committee of The First People’s Hospital of Xuzhou. Signed written informed consents were obtained from all participants before the study.

Patients in the control group were between 25 to 43 yr old (average=30.4±5.6 yr). In the control group, there were 5 cases with less than 28 weeks in their pregnancy and 25 cases were between 28 to 36 weeks. We had 27 cases of partial placenta previa, 3 cases of total placenta previa, 4 primiparas and 26 multiparas in the control group. Patients in the observation group were between 24 to 44 yr old (average=31.2±5.9 yr). There were 4 cases with less than 28 weeks in their pregnancy, and 26 cases were between 28 to 36 weeks. Twenty-nine cases had partial placenta previa and one case had total placenta previa in the observation group. We had five cases of primiparas and 25 multiparas. There was comparability between two groups’ baseline data.

### Nursing method

Specialized nursing was provided for patients in the control group. This method consisted of examination, body position, vaginal bleeding monitoring and recording, psychological consultation and medication observation. Patients rested in their bed to improve placenta’s blood circulation. Left lateral position was recommended to avoid the inferior vena cava being compressed by uterine and to decrease umbilical cord compression. In order to improve the oxygen supply for mother and her baby, intermittent oxygen inhalation was given 3 times a day, and each time lasted 1 hour. We performed body examination and avoided any anal or vaginal examinations. Vaginal bleeding time and vital signs were monitored and recorded. Perineal care was performed twice a day and fetus condition in the uterine was monitored. Pregnant women were told to count fetal movements 3 times a day and one hour each time. The 12 h fetal movement was calculated based on the sum of 3 times movement number multiplied by 4. It was suggested to count fetal movement instead of listening to the fetal heart beat in case of stimulating uterine to induce contraction and monitored fetal heart when it was necessary.

Psychological counseling: we did our best to learn about pregnant women’s emotional state such as their nervousness, anxiety and fear. We explained causes and the prognosis of placenta previa as well as the safety of expectant timely treatment. We also provided comfortable hospitalization environment and carried out educational programs using audio-visual instruments. We sustained normal blood volume and performed blood transfusion when necessary. Patients took hematinic, calcium tablets, Vc and other medications according to the doctor’s orders. Patients were advised to consume foods rich in iron and protein (beans, liver and fresh vegetables). Measures were taken to avoid constipation and proper doses of sedatives, hemostatics and tocolytics were administered. We also administered magnesium sulfate (7.5 g to 25.0 g) to relieve spasm and pain, and 7.2 mg to 14.4 mg salbutamol every day and monitored the heart rate. The abovementioned medications were stopped when the heart rate was over 120 beats per min. Dexamethasone (5 to 10 mg) was injected intramuscularly twice a day for 2 to 3 days in order to promote fetal lung maturation. We terminated the pregnancy when the treatment was adopted after 36 weeks and when every index suggested the maturity of the fetus.

As soon as the condition was judged safe for the mother and her baby, the pregnancy was terminated even in those cases with gestational age of less than 36 weeks. Intrauterine fetal distress condition and profuse bleeding amount were taken into consideration for making this judgment.

Feed-forward control nursing was applied in the observation group. First, we established a feed-forward control nursing improvement team and conducted quality control of nursing defects, put forward improvement idea, and checked the improvement outcome. Feed-forward control nursing improvement team had 10 members and was chaired by the head of nursing department, and comprised of deputy director of nursing department, secretaries, department head nurses and professional nurses with middle and high title. The main duty of this team was to discover any possible nursing defects, offer professional demonstration, supervise timely rectification, and evaluate the effectiveness of the program.

The assessment criteria included professional skills, risk awareness, responsibility education, satisfaction rate, nursing standardized operation and rectification action. Our rectification program proposed risk evaluation questionnaires, which contained questions about mother and baby’s condition, family factor, expectant treatment cognition, expected outcome, pregnant women requirement, nursing manipulation advice, satisfaction and deficiencies. Those items helped us to get a comprehensive knowledge about any possible adverse outcome during the expectant treatment. Efforts were concentrated on issues necessary to assign at least one specialized nurse in charge for every one or two beds, and to arrange flexible shifts to achieve 24 h seamless care to make sure that pregnant women and their family members can have access to nursing services in a timely manner. For pregnant women in emergency, we first performed preliminary assessments under the physician’s judgment. We then carried out specific examination and did our best to avoid any failure caused by excessive manipulation.

Because we were faced with various conditions, we applied layer-management. For the pregnant women with higher education and better compliance, we offered information necessary for diet management, lifestyle, emotion, how to record fetal movement and the best time to report to nurse and doctor. For the pregnant women lacking cognition towards placenta previa and expectant treatment, we strengthened comprehensive education, evaluation work, and talk about any adverse pregnancy event during the treatment and performed a cautious management.

### Observation index

Treatment success rate, complication rate, gestational age, fetal weight, the amount of postpartum bleeding and pregnancy bleeding, nursing service satisfaction score, and nursing error rate were compared between the control group and the observation group.

### Statistical method

SPSS 20.0 (Chicago, IL, USA) was used in this research for statistical analysis. For measurement data, we used mean ±standard deviation. *t*-test was used for comparison between groups . For the count data, we used cases or (%). χ^2^ test was employed for comparison between groups. *P*<0.05 represented statistically significant difference.

## Results

Success rate in the observation group was considerably higher than the control group, while the complication rate was lower in the observation group compared with the control group. Differences were statistically significant (*P*<0.05) (Table 1).

**Table 1:** Comparison between success rate and complication rate (case (%))

**Group**	**Case**	**Success rate**	**Fetal distress**	**Anxiety-depression**	**Puerperal infection**	**Others**	**Total rate**
Control group	30	22(73.3)	3	5	2	1	11(36.7)
Observation group	30	28(93.3)	1	2	1	0	4(13.3)
χ^2^		4.320					4.356
*P*		0.038					0.037

Fetal weight in the observation group increased more than the control group, and the difference was statistically significant (*P*<0.05). The amount of postpartum bleeding and pregnancy bleeding in both groups demonstrated no significant differences (*P*>0.05) (Table 2).

**Table 2:** Comparison among gestational age, fetal weight and the amount of pregnancy and postpartum bleeding

** Group **	** Gestational age (week) **	** Fetal weight(kg) **	** Pregnancy bleeding amount (ml) **	** Postpartum bleeding amount(ml) **
Control group	35.5±1.0	2.8±0.4	120±30	160±35
Observation group	34.7±1.2	2.3±0.5	125±30	165±30
t	4.625	4.958	0.524	0.603
*P*	0.037	0.033	0.748	0.965

Compared to the control group, total satisfaction rate from nursing services was considerably higher in the observation group and nursing error rate was much lower in this group. Differences were statistically significant (*P*<0.05) (Table 3).

**Table 3:** Comparison between nursing satisfaction scale and nursing error rate (case (%))

Group	Case	Satisfied	General	Poor	Total satisfying rate	Discrepancy between nursing and doctor’s order	Discrepancy between infusion and object	Missing and faulty dispensing of drug	Substandard of nursing manipulation	Total error rate
Control group	30	7	12	11	19 (63.3)	4	3	2	4	13 (43.3)
Observation group	30	10	16	4	26 (86.7)	1	1	1	1	4 (13.3)
χ^2^					4.356					6.648
*P*					0.037					0.010

## Discussion

Feed-forward control can eliminate various insecurity factors exist in nursing process and control them prior to the implementation of nursing measures, manipulation of nursing technique and the next nursing process, and to achieve our goals on nursing safety (6).

Nursing quality manifests itself as the sum of the knowledge, professional skills and working attitude of each nurse. Feed-forward control nursing can detect any existing defects and deviant behaviors during the treatment of expectant placenta previa, and analysis the cause of these problems. In addition, it can put forward a rectification plan to correct deviations and prevent any nursing defects, and increase nursing quality (8).

Through detailed analyses, monitoring, evaluation and feedback, we are able to improve nurse’s knowledge as well as their problem-solving skills. Nurses are the first line of caregiving practices in the hospitals (9) and only through strengthening their consciousness about quality management, we can motivate them to actively perform the risk assessment and improve patient’s satisfaction towards clinical nursing work (10).

Patients with placenta precvia are usually emotionally unstable and lack reasoning towards disease development and expectant treatment. They cannot judge their own condition accurately; therefore, they often require more medical care and help (11). If patients feel more comfortable during hospitalization, they are more likely to be satisfied with their caregivers (12). The implementation of a detailed management in the clinical nursing makes the nursing work to start from small goals and gradually move toward bigger goals such as bringing the clinical staff and patient closer to each other. This can also better satisfy patients’ expectations and their diversified health requirements. Besides, through the feed-forward control management, we can complete our department’s nursing security management system, and establish relative regulations and clinical nursing pathway (13), such as major emergency accident, emergency evacuation plan, contingency plan for various types of catheter loss, chemotherapy table and computer input operation process. Therefore, we will be able to change the feedback control of nursing safety into feed-forward control.

Our results showed that the success rate in our observation group was much higher compared to that of the control group. Moreover, complication rate was obviously lower in the observation group. The gestational age and fetal weight improved in the observation group as well. Patients’ satisfaction rate toward nursing services was decidedly higher in the observation group while the rate of nursing errors was considerably lower in this group. The amount of postpartum and pregnancy bleeding did not reveal any meaningful variations.

## Conclusion

Application of feed-forward control nursing model in the expectant treatment of placenta previa can improve treatment success rate, decrease complications and upgrade nursing quality.

## Ethical considerations

Ethical issues (Including plagiarism, informed consent, misconduct, data fabrication and/or falsification, double publication and/or submission, redundancy, etc.) have been completely observed by the authors.
